# Visualizing participant experiences in maternal and child nutrition studies using timeline mapping

**DOI:** 10.12688/gatesopenres.13055.2

**Published:** 2020-06-23

**Authors:** Deepa Sankaran, Priyanshu Sharma, Lisa Lazarus, Tapaswini Swain, Bhanu Pilli, P. Manish Kumar, Vasanthakumar Namasivayam, James Blanchard, Stephen Moses

**Affiliations:** 1 Department of Community Health Sciences, Centre for Global Public Health, University of Manitoba, Winnipeg, Manitoba, R3E0T6, Canada; 2 India Health Action Trust, Lucknow, Uttar Pradesh, 226001, India; 3 Department of Human Nutritional Sciences, University of Manitoba, Winnipeg, Manitoba, R3T2N2, Canada

**Keywords:** Timeline map, maternal and child nutrition, pregnancy, Iron and Folic Acid, implementation research, India

## Abstract

Iron and folic acid (IFA) supplementation is one of the most cost-effective interventions to prevent and treat anemia during pregnancy. Despite having the highest global burden of anemia among pregnant women, rates of IFA uptake in pregnancy in India are still very low, particularly in the state of Uttar Pradesh. While there have been several studies that explored challenges around IFA consumption and adherence, there is a paucity of studies that have synthesized this information into a single visual tool that can help program implementers understand the challenges and identify potential areas of intervention.  Timeline maps were developed as a visual qualitative tool to explore the nuances of health behaviors among pregnant women with respect to antenatal care (ANC) services, including IFA consumption.  Timeline maps were used to visually document critical events pertaining to ANC services chronologically, including details on contact points with the health system and events specific to IFA distribution, consumption and counselling.  Six research assistants (RAs) were trained on how to use timeline maps and record participant narratives. The RAs later participated in a focus group discussion to gain insight about their experiences using the tool. RAs reported that the timeline maps were easy-to-use and facilitated in-depth conversations with participants. RAs shared that they were able to actively engage the participants in co-creating the maps. The visual nature of the tool prompted participants’ recall of key pregnancy events and reflexivity. Challenges reported with the tool/process included recollection of past events and potential misrepresentation of information. These highlight a need to restructure training processes. Our findings indicate that timeline maps have the potential to be used in a variety of other program contexts, and merit further exploration.

## Introduction

India has the highest global burden of iron deficiency anemia in women and children, with the northern state of Uttar Pradesh (UP), India’s largest state, having persistently high rates of anemia in women of reproductive age (52%) and among pregnant women (51%) ^1^. The consequences of anemia during pregnancy are significant, and can lead to poor birth outcomes and postpartum complications ^2, 3^. The National Iron+ Initiative of the Government of India recommends that all pregnant women with hemoglobin levels ≥ 11 g/dl take 180 iron and folic acid (IFA) tablets antenatally, starting at 12–14 weeks of pregnancy, and 180 IFA tablets postpartum ^4^. For pregnant women with hemoglobin levels between 9–11 g/dl, the guidelines recommend taking 2 tablets/day. Distribution of IFA tablets to pregnant women is done through routine antenatal care (ANC) services provided by the Auxiliary Nurse Midwife, a community health practitioner, at designated village health and nutrition days (VHNDs) ^5^. VHNDs are an inter-sectoral convergence platform at the village level, where the community can access a package of services, including registration of pregnant women, ANC services, immunization for all eligible children, growth monitoring, supplementary food provision, and health education. Other frontline workers (FLWs) such as the Accredited Social Health Activist (ASHA) and the Anganwadi worker (AWW) are responsible for follow-up and to ensure distribution of IFA to those women unable to attend VHNDs. Thus, distribution and receipt of IFA tablets are very closely linked to access, availability and quality of ANC services and timely follow-up.

In UP, intake of IFA among pregnant women remains low, with only 12% of pregnant women consuming 100 or more IFA tablets during pregnancy ^1^. Additionally, less than half (46%) of pregnant women have ANC check-ups in the first trimester and only 26% have at least four ANC visits during pregnancy. There are, however, inherent drawbacks to assessing IFA consumption using only quantitative data because of the potential for recall bias, as ANC and IFA data rely on mother’s recall of events up to 5 years before the survey. Several studies that have explored barriers and challenges to IFA compliance highlight different challenges such as poor knowledge of consequences of anemia, accessibility of ANC services, IFA stock-outs, low decision-making power and irregular follow-up by FLWs ^6– 9^. Yet, there is a paucity of studies that have consolidated all of this critical information into a straight-forward, visual tool that can help program managers understand these factors comprehensively and identify potential areas of intervention.

The current study was conducted to explore the feasibility and field-friendliness of timeline mapping to visually document rich narrative data specific to IFA tablet distribution, consumption, counselling and follow-up. The map is intended to enable the implementation team to better understand critical events that occur during pregnancy, and to explore the nuances of health-seeking behaviour among pregnant women with respect to ANC services, including IFA consumption. The study sought to explore field experiences in using timeline maps to facilitate communication with pregnant women, including an understanding of the process of timeline mapping itself.

This feasibility study was part of an implementation study conducted in Behta block of Sitapur district, UP to explore factors associated with IFA consumption during pregnancy and gaps in knowledge of critical pregnancy events from a pregnant woman’s perspective. The implementation study, in turn, is part of larger on-going project based in UP that seeks to reduce morbidity and mortality due to malnutrition in children under five in 25 high priority districts in UP, by strengthening maternal, infant and young child nutrition service delivery through the Government of UP’s Integrated Child Development Services and National Health Mission programs ^10^.

## Developing a timeline map

Narrative interviews are a means of eliciting stories about people’s lived experiences via a conversation between the interviewer and interviewee that allow the interviewer to better understand participants’ experiences ^11^. Participatory visual methods help in shifting power imbalances between the researcher and the participants and provide more meaningful engagement with participants ^12^. Constructing timelines is one way of visually depicting rich narrative data. Timelines are usually constructed from a participant's life events, in some sort of chronological order, with visual notations of highlighted events ^13– 16^. Timelines have been previously used to study several topics including substance use and treatment ^13^, health equity and homelessness ^14^ and resilience among marginalized groups ^15^, but they are still underutilized in maternal and child health and nutrition research. Timelines are a useful tool to explore pregnant women’s antenatal experiences, since pregnancy naturally lends itself to thinking of antenatal experiences in a chronological manner. We used timeline mapping as a methodology, to explore not just the “what” around IFA consumption among pregnant women but also the “why”, within the context of their antenatal experiences.

**Figure 1.**
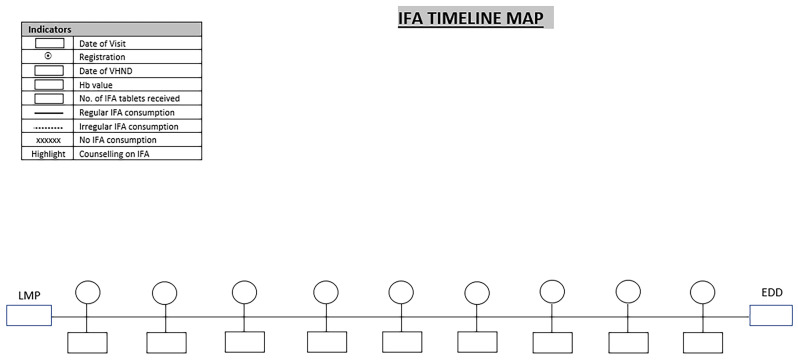
A template of the IFA timeline map. Abbreviations used are: IFA- Iron and folic acid, VHND- Village health and nutrition day, Hb- Hemoglobin, LMP- Last menstrual period, EDD- Expected date of delivery.

The timeline map consists of a straight, horizontal line divided into several compartments ( Figure 1). The circles on top and the squares on the bottom represent the month and date respectively. In our study, timeline maps were used to document (chronologically and in a single visual representation) critical events pertaining to ANC services, different contact points with the health system, and events specific to IFA distribution, consumption and counselling. In this instance, the compartments represented the nine months of pregnancy, and the woman’s last menstrual period and expected date of delivery were used as a frame of reference for the duration of pregnancy ( Figure 1).

The key indicators are highlighted on the top right of the map and serve as a legend for the notations for each indicator. The timeline map is placed on an 11x17 (A3) sheet with sufficient room for the interviewer to annotate key details of participants’ pregnancy events. Having all these details in a single visual representation provides a quick contextual and comprehensive snapshot of all the relevant events from the participants’ perspective. Emphasis is placed on highlighting all the ANC services accessed by the pregnant woman during her pregnancy, including interactions with FLWs. Interviewers are encouraged to probe for details pertaining to each ANC visit such as location, tests performed, receipt of IFA and other supplements. However, the details (including the legend) can be customized to the specific thematic area of the researcher. The timeline map tool and methodology were developed with the assistance of project staff in Kannauj district of UP. 

## Methods

**Figure 2.**
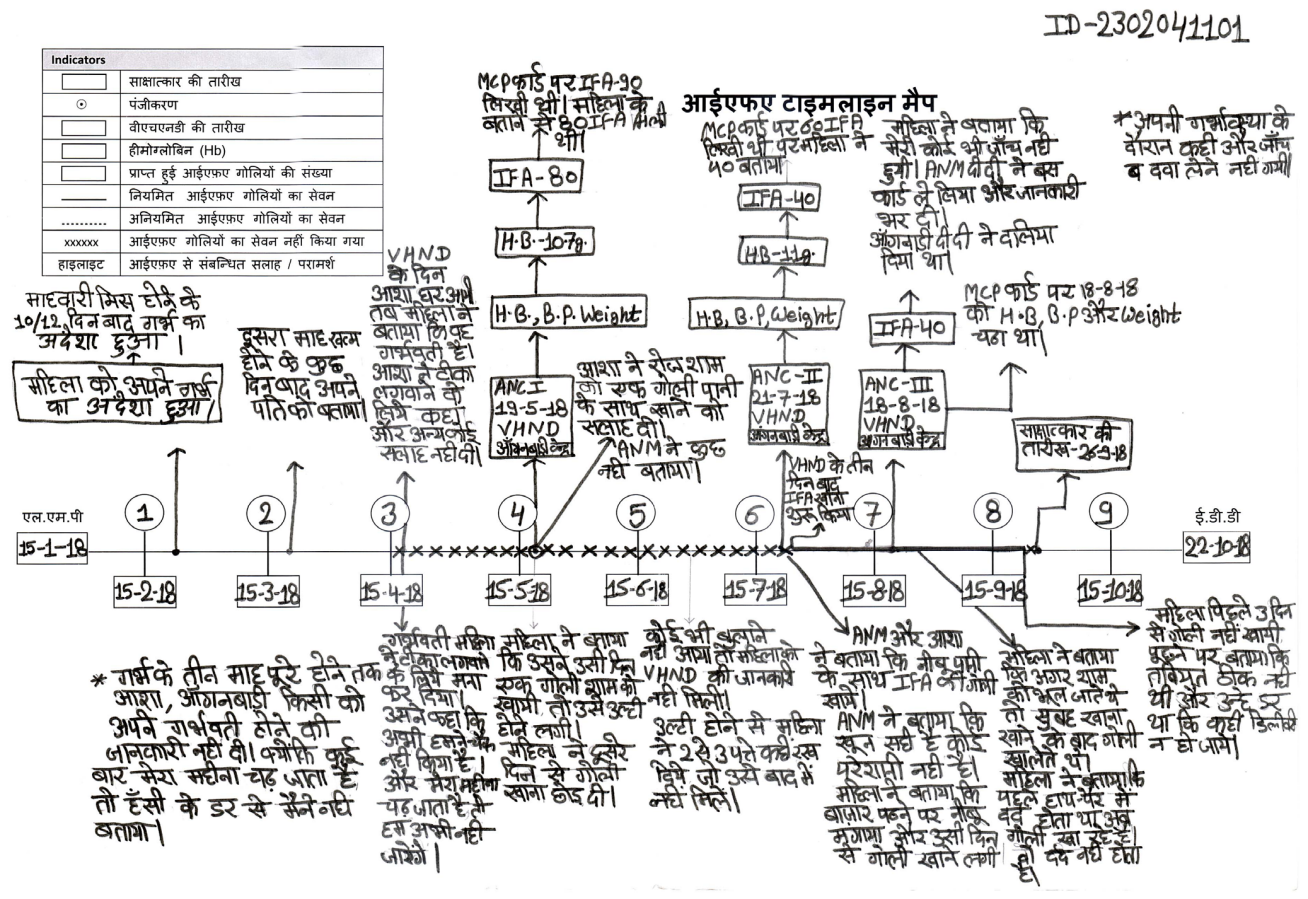
A sample IFA timeline map in Hindi (de-identified). No confidential information is included, and the participant provided written informed consent for sharing de-identified information.

As part of the broader study, in August 2018, in-depth interviews were conducted with fifteen pregnant women in their ninth month of pregnancy in Behta block of Sitapur district in UP to understand the nuances around IFA consumption during pregnancy. Six interviewers (hereafter referred to as Research Assistants, RAs) were selected from among the existing project staff in Sitapur district, as they best understood the project context. The RAs were frontline staff of the nutrition project based at a sub-block level, trained in maternal, infant and young child nutrition as part of the larger project. Their primary responsibility was to mentor and provide on-the-job support to Government of India frontline workers (namely AWWs and ASHAs) to deliver high quality maternal, infant and young child nutrition services. All six RAs had graduate degrees, with more than 2 years of experience working at the community level. The RAs participated in a one-day training led by the project technical experts on the study objectives, how to use the timeline maps and the interview guides. RAs practiced using the tool on each other during the training sessions. Prior to actual data collection, the RAs practiced interviewing at least two pregnant women in their catchment areas using the timeline maps. A debrief was held after their practice sessions to respond to any queries or concerns. Signed informed consent was obtained from the pregnant women prior to the start of the interviews. A data collection team of two RAs conducted the interviews using a semi-structured interview guide and the timeline map. The interview guide comprised open-ended questions on different aspects of ANC, with a focus on IFA consumption. Questions included time of registration of pregnancy, ANC services received, IFA/anemia counselling received, number of IFA tablets received during each ANC visit, and patterns in IFA consumption. Of the two RAs, one acted as the moderator and constructed the timeline map, while the other team member was a note-taker and managed the logistics of the interview. While the data from the interviews with the pregnant women are not presented in this paper, a sample timeline map has been included ( Figure 2).

### Assessing the timeline map

An hour-long focus group discussion was conducted with the six RAs in order to explore their experiences using timeline maps. The focus group discussion was conducted at a Community Health Centre in Behta block by technical experts in the project (co-authors PS and TS). Signed informed consent was obtained from the RAs, after which the focus group discussion was conducted in Hindi and was audio-recorded. The focus group discussion guide comprised open-ended questions on benefits and challenges of using the tool, focusing on the RAs’ experiences. The audio file was transcribed and translated into English by a translator hired by the research team. The first author conducted content and thematic analysis of the transcript (with the support of co-authors PS and TS), paying attention to themes surrounding benefits and challenges of using the tool.

The study received ethics approval from the University of Manitoba’s Health Research Ethics Board and from the Sigma-Institutional Review Board in India.

## Results

Focus Group Discussion with RAs to assess their experience with timeline maps

The findings from the focus group discussion with the RAs are presented under the following categories: 1) aspects of the tool that facilitated data organization; 2) how the tool facilitated a more in-depth understanding of a woman’s pregnancy experiences; and 3) challenges faced while using the tool.

### Facilitating data organization

***Structure of the tool.*** The RAs found the tool easy to use. They reported that the format of the tool, including the legend and boxes, made it easy to annotate and follow the flow of women’s narratives.

*These boxes were good, so that there is no problem in writing the date, otherwise, that gets scattered. There were boxes for months too. The marks that are given, which help us identify points in the timeline, made it easy for me to understand what I have represented where. (RA3)*
*The dots and crosses for IFA, along with the permanent lines, identify whether or not she has been eating [IFA tablets]. ... These indications were very good. For the registration, there was a mark with a circle that helped to identify when the registration had happened. (RA4)*

The RAs also found the structure of the tool to be very helpful. For example, they described as helpful having designated spaces to note down services accessed in the top half of the map, and the pregnant woman’s experiences, such as personal details and side effects, in the bottom half.

*Another thing that was good about it was that on the top, we had to write about the ANC, the VHND, and below, we had to write about whatever the woman told us. So we get to write about the counselling, and all information related to the ANC, the VHND as well as what the woman reveals about herself, in a pre-determined manner. (RA5)*

***Chronology of timeline map.*** RAs noted that the timeline map’s chronology helped guide the sequence of questions asked during the interview, so that there was consistency across all interviews.

*The tool provided a chronological process, about which sequence is to be followed from its starting to its end. This was the best part about the tool that it was in a sequence, guiding us to ask specific questions in a specific order. … Whatever had to be done was available on the paper and I knew what I had to ask the woman at what time. The responses obviously initiated several other questions but the tool kept us bound to the format we have to follow. Otherwise, if I did not have this tool and had been asked to verbally assess the pregnant woman’s situation, I would have forgotten or omitted several things. … But through this tool, I did not forget anything anywhere, irrespective of whether I was talking to the first woman or the third, my questions were the same that revealed respective answers. (RA1)*

Another RA expressed that the timelines prompted them to remember details of the interview even when they reviewed the map much later on.

*What happens because of the mapping is that we get to meet the pregnant women only for a little while. But when you go back to sit and read the paper, it feels as though someone’s entire story from the beginning of her pregnancy, to who she told the first time, where all did she go, is revealed. The indicators show whether or not she took her tablets regularly and when she stopped eating them…(RA5)*

***Snapshot of a woman’s pregnancy.*** The RAs noted that the timeline map provided an overall snapshot of all key events during a woman’s pregnancy “in one place” and contained all the information that “could possibly be” elicited from a pregnant woman. They felt that using the map facilitated dialogue with the participants, who revealed information that otherwise “may never have surfaced”.

*When the pregnant woman started talking, we got to know all the details. Otherwise, it is possible that this information would not have ever surfaced. Because of the timeline map, she has told everything properly, from the beginning of her pregnancy… everything. (RA3)*
*The tool contained all the information that could possibly be extracted from a pregnant woman. When did she become pregnant? When did she get herself registered? When was her ANC done and when she got the iron tablets and what all she ate during her pregnancy, how she took care of herself and who helped her out in her family, giving and taking information from her, all of this was possible to know and understand because of the timeline tool. (RA1)*

***Co-creation of the timeline map with participants.*** RAs were able to co-create the timeline map with the participants, instead of it being a purely researcher-created timeline. They felt that the map increased participant engagement since the participants were the navigators of the entire process.

*The last pregnant woman I met, when I was doing the exercise with the timeline mapping for her, she was actually pointing out to correct what happened in which month. She was a little educated, so she was participating efficiently in telling me about the point at which her ANC happened and when she went to her maternal house or started with her tablets. She was looking at the map and telling me things. (RA4)*
*This happened with me in [name of village]. …when we reached ahead in the sequence, she realized that she wanted to tell us something and took us back to the previous section. (RA1)*

RAs believed that the tool helped the participants reflect on their pregnancy experiences. One RA felt that the visual nature of the tool and filling it out “in front of” the pregnant woman, rather than writing down notes in a notebook or diary, made the information more transparent and helped in greater reflexivity.

*Looking at the map, they would also be amazed at the extent of information that was being taken from them from the beginning until the end of their pregnancy, wondering what would happen with it and realizing that these are important things and whatever they have forgotten or omitted, are actually important and should be followed. …The woman was also able to understand what she followed or omitted. Otherwise, I would just note things in my diary and she would not understand anything. But when I was filling the timeline map in front of her, she could see what was being discussed about her own pregnancy and what were the things she had done wrong or right. (RA3)*

The RAs found that having a pre-determined structure and a sequence to follow helped with consistency across all interviews. Visually summarizing all the information in “one place” provided a snapshot of all relevant pregnancy events. Notably, the visual nature of the timeline map aided in co-creation with the participants, and having the detailed map laid out in front of them also resulted in greater reflexivity among participants.

### Understanding a woman’s pregnancy experiences

***Greater empathy towards participants.*** RAs reported that the timeline mapping exercise provided deeper insight into and sensitivity towards different experiences during a woman’s pregnancy. They felt it helped them empathize with the participants, as opposed to just viewing them as beneficiaries who needed to be counselled. They felt that this also improved their own field work since they had a deeper understanding of pregnant women’s experiences. They reported incorporating insights from the timeline mapping process in their routine home-based counselling of pregnant women.

*Generally, when we conduct home visits, we talk about the state of the month we are visiting in and give the woman counselling based on that. In the case of the timeline mapping, we had to take the information of their entire history. We held the discussion on their entire history and not only that, if there was something wrong or problematic in her history, we did not point it out and start giving advice, we were only trying to understand. …[the timeline mapping exercise] has allowed us to associate with the beneficiary with sensitivity (RA1)*
*The assessment of the pregnant women also helped us in the field. The questions I would otherwise not ask the woman, I have been asking now and talk to them accordingly… (RA3)*

***Eliciting previously ‘unknown’ information.*** RAs shared that the tool helped elicit information from the pregnant women. They felt that it helped reveal the women’s ‘thought processes’, including personal fears and misgivings with respect to revealing their pregnancy, accessing services during pregnancy, unwanted pregnancies and taking IFA.

*Through this [tool], we even got to know that in case she has had one or two miscarriages, she tries to hide her pregnancy because if people find out, she will be made fun of for being pregnant again, after a miscarriage. Women do not reveal their pregnancy to ASHA worker or AWW up to four or five months sometimes, out of fear of being jested at. They do not feel that it is important to reveal this information... (RA4)*
*…When we ask her about why or why not did she did a certain thing, she tells us about her thought process, for instance, that eating the tablet might make her child dark complexioned. Whatever her deepest thoughts are, they are revealed with this tool. (RA2)*
*…after becoming pregnant, women have quietly left for their maternal home and not told anyone… including the husband. And later, when the ‘dawa’ [abortion pills] have not worked, then they come back to their husbands to tell them about it. When we would go for home visits [earlier], such things are not revealed. (RA5)*

***Understanding support systems and key influencers.*** RAs reported getting a better understanding of which family members influenced a pregnant woman’s health behaviors and related decisions.

*Through this map, we even get to know about the family member the woman is closest to and who supports her in all her good and bad times and takes care of her diet, her day-to-day activities and who really pays attention to her in the house or say, which family member, like the mother-in-law, stops her from doing certain things. (RA2)*
*… there are certain families in which the mother-in-law, the father-in-law, husband, all help out. In that family, everyone was helping her out, all medicines, everything. …She was getting a lot of support. (RA4)*

The RAs shared that the narratives that evolved as part of the timeline mapping exercise promoted compassion towards the participants, and greater insight into different aspects that could influence health behaviors. In fact, the Hindi word that the RAs used was *samvedana* which loosely translates to sensitivity/compassion. Additionally, they observed that the tool helped to provide space for the women to share their fears and misgivings, decisions surrounding family upheavals, and feelings on unwanted pregnancies. They also gained an understanding of a pregnant women’s support systems and influencers within their families. RAs had already started incorporating insights from the timeline mapping process in their routine home-based counselling.

### Challenges faced while using timeline maps

When asked about some of the challenges of using the tool and suggestions to improve the tool and the interview process, several of the RAs noted that some of the participants may have misrepresented, misremembered or felt uncomfortable answering questions (one-on-one) about their pregnancy. They suggested that having another family member present during the interview, particularly someone who was close to the pregnant woman, would alleviate this.

*…along with involving the beneficiary, we should also involve the one person who is the closest to her and spends maximum time with her. If we take their view as well, then we will be able to understand the situation even better. (RA1)*
*…When I just spoke to the pregnant woman, she told me that she has been eating everything. I would have written everything down since I was only talking to her. But her mother in law joined us in between and told us that she had not eaten anything, she even showed me the entire bottle of syrup [iron supplement] still full, the powder had also dried up. So, if I just speak to the pregnant woman, some of them may lie and that cannot be detected. (RA5)*

Another challenge that one RA faced was participants being hesitant to provide information. This RA also felt that since the respondents were in the ninth month of their pregnancy, recollection of past events was somewhat of a challenge at the beginning of the interview. However, once the interview progressed, this posed less of a challenge.

*In the beginning, when we went and sat with them and talked a little, they would initially get a little scared looking at the timeline map, wondering what it had to do with their pregnancy. They were hesitant to answer a few initial questions. Say, it is someone’s ninth month into pregnancy, they would have to recall things from nine months ago, about when they had their last menstrual cycle, who they told about the pregnancy… But after we were able to extract information about a month or two, and reach up to the timeline of one ANC, they would open up, discuss amongst each other and speak on their own, telling us about everything that happened. Yes, recollection was a little challenge. (RA4)*

Some RAs suggested, as a means to improve the tool, having pre-drawn boxes for when pregnant women should have their four ANC check-ups. They conjectured that doing so would increase participant involvement and would help the pregnant woman ‘realize’ that she should have had her ANC check-up during the recommended time period.

*There should be boxes made for the ANC. What this will do is that if there are four ANCs and four boxes at the right places, we can show that to the woman and in case her ANC did not happen in time, we can show her where she has missed out on things. …(RA2)*
*… [if there were preset boxes] she will know that the ANC visit was supposed to happen but did not; this will get her more involved in the mapping. With the help of the box, she will get to know what the actual position should have been and why [I am] marking it with an arrow. She will understand it better. (RA3)*

While discussing challenges with the tool/process, the RAs suggested that having another family member present during the interview would alleviate the participants’ discomfort and potential misrepresentation of information. They also observed that while, initially, recollection was a challenge for a few participants since they were being asked about events that occurred a while ago, it was less of an issue as the interview progressed. RAs also recommended making certain structural changes to the tool to enhance researcher and participant experiences.

## Discussion

This study sought to describe and understand the use of the timeline map as a qualitative visual tool within an implementation research context. The findings suggest that the timeline map has great potential as a participatory, narrative interview tool to provide an in-depth understanding of women’s pregnancy experiences.

There are several reasons why the interviewers felt that the narrative interview process was made easier by using the timeline map. The structure of the tool helped organize their thinking around the interview process and helped them be consistent across interviews. The visual nature of the maps likely serves as a more effective guide, compared to a traditional discussion guide, to interviewers who may be less experienced as well. Nonetheless, RAs also reported using the semi-structured interview guide as a checklist during the interviews to ensure they did not inadvertently omit any key topics. Stewart-Tufescu and colleagues ^17^ described how the “visibility” of a graphic interview tool helped participants or researchers return to a topic, as compared to a standard narrative interviewing technique. The RAs also observed that participants would, at times, point to a section that had already been discussed and add to the details. They also posited that the tool encouraged greater participation and reflexivity among the participants. Several studies have outlined how visual methods are more participatory and provide a more holistic view of the topics being researched ^16– 19^. Additionally, participatory visual methods help in shifting power imbalances between the researcher and the participants and provide more meaningful engagement with participants ^12^.

In our study, the timeline map was intended as a means to organize complex narrative data. However, the RAs found that it also served as a means to reveal information that was previously not known and encouraged pregnant women to explore their pregnancy experiences and reflect on them. Visual methods, such as timelines, when combined with interviews, focus “attention beyond what is possible through talk alone, thus becoming not only a piece of data in its own right but a vehicle through which further data were produced” ^16– 20^.

We chose to train existing project staff as RAs, instead of using external researchers, since they best understood the project context. The tool’s easy-to-use interface is well-suited for program managers and staff to administer. The advantages to this approach were that the project staff were familiar with the implementation topic being studied; they were experts in rapport-building, since their responsibilities in the wider project entailed extensive nutrition counselling; and it provided them with a better understanding of all the events around a woman’s pregnancy. Moreover, an unintended consequence was that the RAs reported developing greater sensitivity and empathy towards women’s experiences during their pregnancies. For instance, they reported understanding why some women had misgivings about revealing their pregnancy to FLWs and family members, or why they did not attend ANC clinics. RAs revealed that the timeline exercise gave them greater insight into aspects of women’s pregnancies that could affect pregnancy-related health behaviors, particularly deeply personal ones such as family upheavals and unwanted pregnancies. Internal empathetic validity is the potential of research to transform the emotional dispositions of researchers and participants towards each other, in a manner that promotes interpersonal understanding and compassion ^21^. The implications of this are enormous, particularly in relation to knowledge translation. Greater interpersonal understanding between researchers and knowledge users promotes enhanced engagement in producing and applying knowledge by both parties ^22^. Indeed, the RAs noted that participating in the timeline mapping exercise changed how they approached interpersonal counselling when visiting the homes of pregnant women, and that they were now incorporating questions that they “previously did not ask”.

The RAs noted some challenges with the tool/process. They felt that some participants misrepresented or misremembered information pertaining to their pregnancies and suggested that having another family member present during the interview could alleviate this problem. However, this could lead to other biases in reporting, and could inhibit honest responses from participants. The RAs’ suggestion of having another family member to “fact-check” participants’ responses suggests a need for more interviewer training, focusing on the nuances of probing and follow-up questions. Recollection of events was identified as a challenge for a few participants, at least initially, since they were being asked about events that occurred some time ago. However, the RAs observed that it was less of an issue as the interview progressed. Prolonged participant engagement, using multiple methods of data collection and participant validation are some ways to enhance trustworthiness of findings using temporal visual methods such as life histories and timelines ^23^. RAs also recommended adding more “ANC boxes” to the tool to enable better notations, and to serve as a visual reminder to participants that accessing ANC services in the recommended time period was crucial.

This study has several limitations. The small number of interviewers provided a limited perspective on usability and accetability of the tool. Another limitation is that this paper only presents interviewers’ experiences and does not capture participants’ experiences with the tool. Power imbalances between the FGD moderators (co-authors PS and TS) and the RAs could be a potential limitation, since the moderators were the senior technical experts in the project. The RAs may have felt that they had to speak positively about the tool. However, the advantages of having the co-authors as the FGD moderators was the technical expertise that they possessed and the pre-existing rapport that they had with the RAs. Both FGD moderators (PS and TS) were aware of the power inequalities and tried to mitigate them in a number of ways. For instance, the Community Health Centre where the FGD was conducted was collectively decided upon by the RAs and the moderators, being mindful of the distances the RAs had to travel. The moderators opened the discussion by stating that the purpose of the FGD was to understand RAs’ experiences using the tool and by encouraging the RAs to provide inputs on how to improve it. By restating this often and by offering encouraging comments throughout the FGD, the moderators may have contributed to a safe atmosphere. Furthermore, the fact that the RAs shared challenges using the tool points to them feeling comfortable reflecting on their experiences using the map.

Despite the visual nature of the tool, participant literacy could pose a limitation. We did not systematically address the effect of literacy on the level of participation in the interviews. However, RAs interviewed an almost equal number of illiterate and literate women. According to the RAs, the level of participation was similar across participants because of the visual nature and structure of the timeline map. Due to the tool’s visual nature, the participants could understand the sequence of events being noted down which kept the conversation going. Visual methods, when combined with interviews, enhance participant engagement and facilitate a more in-depth exploration of experiences, possibly because they serve as a common visible frame of reference for the discussion between the interviewer and the interviewee ^16, 24^. This is of particular significance in overcoming linguistic and literacy barriers.

In this study, we have outlined the feasibility and field-friendliness of using timeline maps, in an implementation research context, to understand in greater detail the key events during pregnancy and how these events influenced IFA consumption. Timeline mapping is an alternate method to understanding the complexities around IFA consumption in pregnancy, thus circumventing biases inherent in more traditional, self-reported methods of IFA assessment. It can be a valuable tool for understanding other events that change over time as well. Within the domain of maternal, neonatal and child nutrition, for instance, it can be used to explore complementary feeding practices among children 6–23 months of age, understanding events that influence early initiation of breastfeeding in a labor room, and exploring food behaviors of new immigrants. Timeline maps can also be used as a training tool and for nutrition counselling where frontline workers could use an ‘ideal’ timeline map depicting all the health services available to pregnant women during the course of their pregnancy to create greater awareness in the community. These alternate uses might be tested in future studies.

## Conclusion

The timeline mapping tool is an easy-to-use and effective participatory tool that can be included in implementation research and program evaluation. It has immense potential to be used widely in studying other areas of program implementation. The visual representation of key pregnancy-related events, including service delivery at different time points, provides an opportunity address program gaps and to improve program delivery.

## Data availability

For confidentiality reasons, the transcript of the focus group is not available; the focus group discussion was with six individuals who are still employed with the project. It should be noted that all relevant aspects of the focus group discussion have already been included in the article as quotes.
