# Supplementary material for: Visualizing participant experiences in maternal and child nutrition studies using timeline mapping
Source: Gates Open Res. 2020 Jun 23;3:1535. Originally published 2019 Sep 11. [Version 2] doi: 10.12688/gatesopenres.13055.2 (PMC7343969; doi:10.12688/gatesopenres.13055.2)
Supplement: Peer review history

# Peer review: Reviewer response for version 2

**DOI:** 10.21956/gatesopenres.14343.r28982

**Published:** 2020-07-08

**Authors:** Eva C. Monterrosa

**Affiliations:** 1 Global Alliance for Improved Nutrition, Basel, Switzerland

The revisions are appropriate and the article is greatly improved. I approve this manuscript for indexing.

Is the rationale for developing the new method (or application) clearly explained?

Partly

Is the description of the method technically sound?

Partly

Are the conclusions about the method and its performance adequately supported by the findings presented in the article?

Partly

If any results are presented, are all the source data underlying the results available to ensure full reproducibility?

Partly

Are sufficient details provided to allow replication of the method development and its use by others?

Partly

Reviewer Expertise:

NA

I confirm that I have read this submission and believe that I have an appropriate level of expertise to confirm that it is of an acceptable scientific standard.

# Peer review: Reviewer response for version 2

**DOI:** 10.21956/gatesopenres.14343.r28981

**Published:** 2020-07-02

**Authors:** Rebecca Heidkamp

**Affiliations:** 1 Department of International Health, Johns Hopkins Bloomberg School of Public Health, Baltimore, MD, USA

The authors have addressed most of the concerns raised by reviewers. This methods-focused paper will be useful to researchers and practitioners alike.

Is the rationale for developing the new method (or application) clearly explained?

Partly

Is the description of the method technically sound?

Partly

Are the conclusions about the method and its performance adequately supported by the findings presented in the article?

Partly

If any results are presented, are all the source data underlying the results available to ensure full reproducibility?

No source data required

Are sufficient details provided to allow replication of the method development and its use by others?

Partly

Reviewer Expertise:

large-scale evaluation, household survey measurement, maternal child nutrition

I confirm that I have read this submission and believe that I have an appropriate level of expertise to confirm that it is of an acceptable scientific standard.

# Peer review: Reviewer response for version 1

**DOI:** 10.21956/gatesopenres.14183.r28344

**Published:** 2020-01-20

**Authors:** Rebecca Heidkamp

**Affiliations:** 1 Department of International Health, Johns Hopkins Bloomberg School of Public Health, Baltimore, MD, USA

This article presents an overview of a small pilot study that examines the experience of 6 research assistants in India around using a "timeline mapping" tool during interviews with a total of 15 women in their 9th month of pregnancy. The tool is designed to elucidate a clearer picture of the woman's experience of care during the pregnancy (Including ANC and IFA). The authors used Focus Group Discussions to better understand the Research Assistant's experience of the tool.

 I really appreciate that the authors are profiling an innovative tool to facilitate qualitative data collection and in this instance, to help improve our understanding of women's ANC experiences. Low IFA intake is a global problem, as are the limitations in the approaches we have used to try to asses and improve compliance. 

 The manuscript can be improved by addressing the following issues:

 Introduction

- The specific objective of this study is not clear as it is currently framed as "exploring the use" of the map as a way to inform program implementation.  However, the latter aspect does not appear to be addressed. Given that the "results" section is focused on findings from FGDs with the 6 RA, the aims should be more clearly explained in terms of assessing the RA experience with the tool.
- The majority of background in both the abstract and paper is about  low uptake of IFA however as presented, the results and discussion are not focused on IFA. Consider reframing background or more explicitly addressing IFA in other sections.
- If IFA is going to remain the focus then it's important to acknowledge the issues with measurement validity when coverage of intake is measured using HH surveys & extended recalls about specific number of pills - the 12% intake of 100+ is likely based on recall that is highly subject to bias.

 Methods

- My expertise is not in qualitative methods but encourage authors to consider comments by Dr Monterrosa.
- The FGD appears to be the core of the study in terms of the results reported. Considering changing the subtitle before the FGD paragraph to be more descriptive e.g. "FGD with RA to assess their experience with the map"

 Conclusions

- Authors state "In this study, we used timeline mapping to understand in greater detail the key events during pregnancy and how these events influenced IFA consumption, from a pregnant woman’s perspective."  However these are not the results presented. Rather the results are related to the RA experience using map.  This ties back to comments about the introduction and needing to be clearer about the actual aims. 

Is the rationale for developing the new method (or application) clearly explained?

Partly

Is the description of the method technically sound?

Partly

Are the conclusions about the method and its performance adequately supported by the findings presented in the article?

Partly

If any results are presented, are all the source data underlying the results available to ensure full reproducibility?

No source data required

Are sufficient details provided to allow replication of the method development and its use by others?

Partly

Reviewer Expertise:

large-scale evaluation, household survey measurement, maternal child nutrition

I confirm that I have read this submission and believe that I have an appropriate level of expertise to confirm that it is of an acceptable scientific standard, however I have significant reservations, as outlined above.

# Response

**Published:** 2020-06-15

**Authors:** Deepa Sankaran

**Affiliations:** University of Manitoba, Canada

**Introduction**

- The specific objective of this study is not clear as it is currently framed as "exploring the use" of the map as a way to inform program implementation.  However, the latter aspect does not appear to be addressed. Given that the "results" section is focused on findings from FGDs with the 6 RA, the aims should be more clearly explained in terms of assessing the RA experience with the tool.

 

 Response: We appreciate the reviewer's comments. This paper sought to describe the feasibility and acceptability of a novel qualitative data visualization tool, as part of a larger implementation study seeking to improve iron and folic acid tablet adherence among pregnant women. For further clarity, we have modified the objectives of the study as stated on Pg. 5 (2 ^nd^ paragraph)

  

- The majority of background in both the abstract and paper is about low uptake of IFA however as presented, the results and discussion are not focused on IFA. Consider reframing background or more explicitly addressing IFA in other sections.

Response: We appreciate the reviewer’s suggestion of reframing the background, or addressing IFA in other sections. The intent of this paper was to focus on a novel methodology of obtaining general IFA consumption patterns, which is particularly salient given measurement validity issues with quantitative IFA intake measurements. We have split the introduction into two parts. The first part, “Introduction” (Pg 4-5), provides background on why understanding IFA consumption from a woman’s perspective is key. The second part, “Developing a Timeline Map” (Pg 6-7), provides background on the use of visual methods in qualitative data collection. We intend to present the results from the timeline maps in an upcoming publication, where we specifically outline the IFA consumption patterns obtained using timeline maps.

  

- If IFA is going to remain the focus then it's important to acknowledge the issues with measurement validity when coverage of intake is measured using HH surveys & extended recalls about specific number of pills - the 12% intake of 100+ is likely based on recall that is highly subject to bias.

Response: We have addressed (Introduction, Pg 4, 2 ^nd^ paragraph) the inherent recall bias associated with all quantitative data with respect to ANC and IFA, since most surveys rely on mother’s recall of this information, up to 5 years prior to the survey.

**Methods**

- The FGD appears to be the core of the study in terms of the results reported. Considering changing the subtitle before the FGD paragraph to be more descriptive e.g. "FGD with RA to assess their experience with the map"

Response: Thank you for this suggestion. Please see changes made to Results section (Pg 8) to include the subtitle “Focus Group Discussion with RAs to assess their experience with timeline maps”.

**Conclusions**

- Authors state "In this study, we used timeline mapping to understand in greater detail the key events during pregnancy and how these events influenced IFA consumption, from a pregnant woman’s perspective."  However, these are not the results presented. Rather the results are related to the RA experience using map.  This ties back to comments about the introduction and needing to be clearer about the actual aims.

Response : We appreciate these comments. Please see changes made on Pg 15 (last paragraph) to better reflect the aims of this study.

# Peer review: Reviewer response for version 1

**DOI:** 10.21956/gatesopenres.14183.r27817

**Published:** 2019-10-21

**Authors:** Eva C. Monterrosa

**Affiliations:** 1 Global Alliance for Improved Nutrition, Basel, Switzerland

I would like to thank the Authors for proactively seeking to examine the feasibility of a qualitative data collection tool. This type of research is critically needed to advance implementation science in nutrition. To strengthen this paper, I recommend the following changes before this manuscript is indexed.

Introduction

- Clarity of objective. Please define what you mean by implementation study. Is this a feasibility study to assess its usability for data collection and analysis to achieve your objectives? Is this an exploratory study to examine the acceptability (e.g.,field-friendliness) of tool among field staff doing qualitative research?
- Please further elaborate on the context for the research methodology. For example, the authors might want to consider the skill level needed to conduct high quality interviews and how this tool might provide the structure that a discussion guide might lack for a less experienced staff. Another good point to make is that participatory methods not only support data collection through reflexivity but also empower participants.

Methods

- The timeline is a tool to construct a chronological narrative of events – something the authors have noted. As such, it is both an elicitation technique for constructing event-based narratives organized in chronological order, as well as a data visualization tool because text data is made visible to the interviewee in a way that interviewer notes or voice recordings are not. Please distinguish this point in the methods section. The articles cited in the methods are a combination of the timeline method and graphic elicitation which is the use of drawings, pictures or symbols to tell stories about events and to construct deeper, richer narratives/meanings.
    
  1. I’m having trouble seeing how the present tool is a graphic elicitation technique. If yes, please clarify/justify how the graphics (squares, circles, lines) helped with constructing a richer, deeper narrative that would not have been possible through a standard interview guide? Otherwise I think what we have is a data visualization tool and this distinction needs to be clear.
  2. Would illiterate women be as reflexive of their personal experiences with the map you have constructed, which is text heavy? If all women were literate, please add this as a limitation of your study or generalizability of your results only to this group of women.

- Please describe more fully who the RAs were? What other jobs were they working? Did any of them have qualitative research experience?
- How long, on average, did the interview last?
- Who were the participants – age, education, income bracket.

 Results

- The results section needs a bit of tightening so that it aligns more closely with the revised objectives. For example, I don’t know why certain ‘background’ information was included because it does not seem relevant for the replicability and generalizability of this tool. E.g., ‘unknown’ information & Understanding support systems and key influencers sections. Does this relate to the fact that they also have a job as nutrition counsellors? The support system info would have been retrieved in a standard interview.
- How much did they use the semi-structured interview guide? Were the questions even needed?
- For each RA, how many of timeline maps did they need to do to feel comfortable with using the tool? (i.e., what was the learning curve?)
- Some of the results/recommended changes to do not align with improving the tool, such as: 
   
  1. having the MIL in the room while PW is interviewing
  2. Pre-drawn boxes for when pregnant women should have their four ANC check-ups. This would bias the tool.

- It wasn’t clear if these were one-on-one interviews or group interviews. Please check the text because in some places it seems that group interviews are implied (i.e., ‘they would open up, discuss amongst each other and speak on their own’…)

 

Discussion

- Please discuss the overall quality of the interview with regards to having achieved your objective. Did the researchers retrieve better information?
- Presence of social desirability - RAs noted that some of the participants may have misrepresented, misremembered. This idea needs to be better outlined in the discussion section. How do qualitative researchers address validity of the findings?
- Please comment how this tool might have alternative application to research. For example it could be used by front line staff to deliver nutrition counselling and to support greater client empathy. This use might be tested in future studies.
- Check for repetition in text.

Is the rationale for developing the new method (or application) clearly explained?

Partly

Is the description of the method technically sound?

Partly

Are the conclusions about the method and its performance adequately supported by the findings presented in the article?

Partly

If any results are presented, are all the source data underlying the results available to ensure full reproducibility?

Partly

Are sufficient details provided to allow replication of the method development and its use by others?

Partly

Reviewer Expertise:

Qualitative research; maternal and child nutrition; India

I confirm that I have read this submission and believe that I have an appropriate level of expertise to confirm that it is of an acceptable scientific standard, however I have significant reservations, as outlined above.

# Response

**Published:** 2020-06-15

**Authors:** Deepa Sankaran

**Affiliations:** University of Manitoba, Canada

Introduction

- Clarity of objective. Please define what you mean by implementation study. Is this a feasibility study to assess its usability for data collection and analysis to achieve your objectives? Is this an exploratory study to examine the acceptability (e.g. field-friendliness) of tool among field staff doing qualitative research?

Response: Thank you for this query. This paper sought to describe the feasibility and acceptability of a novel qualitative data visualization tool, as part of a larger implementation study seeking to improve iron and folic acid tablet adherence among pregnant women. For further clarity, we have modified the objective as stated in the Introduction (Pg. 5, 2 ^nd^ paragraph).

  

- Please further elaborate on the context for the research methodology. For example, the authors might want to consider the skill level needed to conduct high quality interviews and how this tool might provide the structure that a discussion guide might lack for a less experienced staff. Another good point to make is that participatory methods not only support data collection through reflexivity but also empower participants.

 

 Response: We appreciate these suggestions and have included these points in the publication in several places (Pg.6, 1 ^st^ paragraph; Pg. 7, 2 ^nd^ paragraph in Methods; Pg.13, 2 ^nd^ paragraph in Discussion)

Methods

  

- I’m having trouble seeing how the present tool is a graphic elicitation technique. If yes, please clarify/justify how the graphics (squares, circles, lines) helped with constructing a richer, deeper narrative that would not have been possible through a standard interview guide? Otherwise I think what we have is a data visualization tool and this distinction needs to be clear.

Response: In our paper, we sought to describe timeline mapping as a qualitative visualization tool using a pre-prepared diagram. RAs shared that the tool helped guide the participants’ narrative since they could see boxes with information being filled out right in front of them. As such, we agree that the tool functions as a data visualization tool, and not a graphic elicitation tool. We acknowledge that some of the references cited in the discussion section could be misleading. We have edited this section to reflect that timeline mapping is a qualitative visualization tool on pages 13-14 (References in the 1 ^st^ and 2 ^nd^ paragraph of the Discussion).

  

- Would illiterate women be as reflexive of their personal experiences with the map you have constructed, which is text heavy? If all women were literate, please add this as a limitation of your study or generalizability of your results only to this group of women.

 

 Response: We have included some information on participant literacy in the discussion section (pg 15, 2 ^nd^ paragraph) and re-organized previous sections to further clarify the effect of literacy on participation. Briefly, there was an almost equal ratio of literacy levels among women who were interviewed. According to the RAs, the level of participation was similar across participants because of the visual nature and structure of the timeline map. Due to the tool’s visual nature, the participants could understand the sequence of events being noted down which kept the conversation going.

- Please describe more fully who the RAs were? What other jobs were they working? Did any of them have qualitative research experience?

Response: As suggested, we have included a description of the RAs in the methods section (pg 7, 1 ^st^ and 2 ^nd^ paragraph in Methods). Briefly, the RAs were frontline staff of the nutrition project based at a sub-block level. Their primary responsibility was to mentor and provide on-the-job support to Government of India frontline workers (namely the Anganwadi workers and the ASHAs) to deliver high quality maternal, infant and young child nutrition services. All six RAs were post-graduate, having more than 2 years of experience in working at the community level. They are trained in Maternal, Infant and Young Child Nutrition in the larger project.

**Results**

  

- How much did they use the semi-structured interview guide? Were the questions even needed?

 

 Response: We have included a description of how the interview guides were used on Page 13, 2 ^nd^ paragraph in Discussion. Briefly, RAs reported using the interview guides in all the interviews. The guide was created to ensure that the RAs asked participants key questions around ANC care-seeking, pregnancy and IFA receipt and consumption. RAs also reported using the interview guide as a checklist during the interviews to ensure they did not inadvertently omit any of these key questions. However, RAs were trained to encourage participants to narrate their experiences around a certain topic, for instance, the timing of their first ANC check-up.

    

- For each RA, how many of timeline maps did they need to do to feel comfortable with using the tool? (i.e., what was the learning curve?)

Response: During the in-class training sessions, all RAs took turns interviewing one another using the interview guides and practiced filling out the timeline maps. After this, prior to actual data collection, the RAs practiced interviewing at least two pregnant women in their catchment areas using the timeline maps. A debrief was held after their practice sessions to respond to any queries or concerns. We’ve revised our methods to include this information (Pg.7, 2 ^nd^ paragraph in Methods).

Discussion

- Please discuss the overall quality of the interview with regards to having achieved your objective. Did the researchers retrieve better information?

 

 Response: With respect to the quality of the information retrieved, using the timeline maps produced more nuanced information which ultimately helped design program implementation strategies to improve IFA consumption among pregnant woman (data not shown). With respect to interviewers’ skills, this has been addressed on Pg 13 (2 ^nd^ paragraph in Discussion). We explored using project staff as interviewers for the first time. Project staff were already experts in rapport-building, which plays a key role during the interview process. It also had the unintended consequence of making our project staff more empathetic and compassionate in their routine counselling activities. We have not addressed this per se in the discussion since we do not present any data from the timeline maps. However, we have re-iterated in the discussion (Pg. 15 last paragraph) that this study explored field-friendliness of timeline maps.

  

- Presence of social desirability - RAs noted that some of the participants may have misrepresented, misremembered. This idea needs to be better outlined in the discussion section. How do qualitative researchers address validity of the findings?

 

 Response: This has been expanded upon in the discussion (Pg 14, last paragraph) with respect to how visual methods can address validity/trustworthiness of their findings.

  

- Please comment how this tool might have alternative application to research. For example, it could be used by front line staff to deliver nutrition counselling and to support greater client empathy. This use might be tested in future studies.

 

 Response: We appreciate the reviewer’s suggestion of including alternative applications of the tool in the future. When this feedback was taken back to the RAs, they came up with a number of recommendations for future use. They suggested using the tool for nutrition counselling where frontline workers could use an ‘ideal’ timeline map depicting all the health services that pregnant women should receive during the course of their pregnancy to create greater awareness in the community. They also suggested using it as a training tool while training/mentoring Government of India frontline workers. Please see Pg.17 (last paragraph) for edits.
